# Editorial: Innovative value-based medicine: lessons from China's healthcare evolution

**DOI:** 10.3389/fpubh.2026.1783125

**Published:** 2026-01-26

**Authors:** Gang Lv, Xiangxiang Jiang, Jing Yuan, Z. Kevin Lu

**Affiliations:** 1 Department of General Surgery, The First Medical Center of Chinese PLA General Hospital, Beijing, China; 2 Department of Clinical Pharmacy and Outcomes Sciences, College of Pharmacy, University of South Carolina, Columbia, SC, United States; 3 Institute of Chinese Medical Sciences, University of Macau, Taipa, Macau, China

**Keywords:** China, cost-effectiveness, health policy, health system reform, value-based medicine

Healthcare systems face a dual challenge of ensuring access to innovations while preserving financial sustainability, giving rise to value-based medicine (VBM), which balances clinical benefit and cost to optimize population health outcomes (1). Additionally, VBM aligns incentives among payers, providers, and patients by emphasizing outcomes, efficiency, and sustainability (1).

China offers a compelling case study in the global evolution of VBM (Table 1). Over the past decade, China has piloted strategies to balance innovation with sustainable financing, including reforms in hospital governance (2), introduction of novel drug payment mechanisms (2), and broader adoption of big data and artificial intelligence (AI) for monitoring costs and clinical outcomes. (3) These reforms highlight how large health systems apply VBM under demographic change, chronic disease growth, and equity demands.

**Table 1:** Summary of studies on value-based medicine (VBM) in China.

**Study**	**Focus**	**Study design**	**Main findings**	**VBM implications**
Zuo et al.	Hospitalization patterns and costs in cross-border medical tourism for knee replacement	Retrospective cohort study using inpatient record data (2023–2024)	Cross-border patients had shorter length of stay and lower hospitalization costs; length of stay fully mediated cost differences, indicating higher care efficiency	Cross-border care integration may enhance value-based care by improving efficiency, reducing unnecessary resource use, and optimizing costs without compromising outcomes
Guanyi et al.	Relationship between buyer market power, spatial spillover effects, and pharmaceutical industry profitability in China	Empirical analysis using a spatial Durbin model on industry data (2001–2021)	Local buyer market power reduces local pharmaceutical firm profitability and exerts weak negative spillover effects on neighboring regions; supplier counter-power and firm size mitigate some impacts	The VBM policies should align purchasing power with performance-based pricing to sustain efficiency, competition, and long-term value creation
Zhao et al.	Determinants of primary care physicians' intention to provide breast cancer screening services for rural women	Cross-sectional survey of 1,101 primary care physicians using an extended theory of planned behavior model	Subjective norms most strongly predicted intention to provide screening, followed by attitudes and perceived behavioral control; screening knowledge and past behavior also influenced intentions indirectly	Supporting primary care physicians through training, social norms, and enabling resources can increase preventive screening uptake and improve value-based outcomes in underserved populations
Zhang H. et al.	Cost-effectiveness of durvalumab consolidation therapy after chemoradiation for stage III small-cell lung cancer in China	Markov model cost-utility analysis using data from the Phase III trial	Durvalumab improved survival but was not cost-effective at current prices in China, with ICER far above WTP thresholds; sensitivity emphasized drug price as key driver of economics	Balancing clinical benefit with price and reimbursement strategies is crucial to deliver high-value cancer care and ensure innovative therapies provide both health and economic value
Chen et al.	DRG payment reform and costs of lumbar disc herniation	Quasi-experimental (pre-post comparison) design using hospital inpatient records (2017–2022)	The DRG reform is associated with lower hospitalization costs and reduced costs for both Chinese medicine and Western medicine	True VBM in TCM hospitals requires linking payments to outcomes and tailoring reimbursement models to TCM's unique treatment characteristics
Zhang X. et al.	The optimal strategies for supplementing MAs with Internet-based healthcare	A two-stage queuing game-theoretic model	Supplementing MAs with internet hospitals can increase downward referral volume and provider effort under favorable cost, arrival rate, and revenue-sharing conditions, especially within tightly integrated alliances	Integrating digital health platforms into alliance networks can enhance value-based care by improving flow efficiency and resource allocation
Jiang et al.	The effect of China's NVBP policy exclusively for insulin	An interrupted time series analysis using procurement data (2020–2024)	NVBP policy significantly reduced insulin expenditure while improving treatment accessibility and affordability for insulin	NVBP advances VBM by reducing insulin costs and improving access while encouraging the use of cost-effective, higher-value therapies
Wang et al.	Cost-effectiveness of Xianling Gubao Capsules compared to Jintiange Capsules and non-treatment for postmenopausal osteoporosis	Markov model-based cost-effectiveness analysis using data from a network meta-analysis	The ICERs for the Jintiange capsule compared to the Xianling Gubao capsule ranged from $11,955 per QALY at age 55 to $9,711 per QALY at age 74	Integrating Chinese patent medicines supports VBM by emphasizing cost-effective treatments that improve patient outcomes and optimize healthcare resource use
Xu Y. et al.	Developing a standardized MCDA framework tailored for implantable medical devices in China	A mixed-methods design combining a discrete choice experiment and MCDA	Clinical safety (35.45%) and cost (27.94%) are the top-ranked criteria for implantable medical devices	Using MCDA framework can help guide implantable medical device evaluations toward transparent, evidence-driven, and outcome-oriented resource allocation decisions
Wu et al.	Service capacity of secondary hospitals in Guangzhou	Multi-method empirical design	Capacity improved overall, but disparities widened; shift to bimodal distribution	Governance and resource allocation must address efficiency and equity across tiers
Li	MAH system and ESG of pharmaceutical firms	Difference-in-differences using panel data (2012–2019)	MAH enhanced ESG via R&D, pay equity, supply chain; strongest in non-SOEs	Institutional innovation can align corporate behavior with sustainable health goals
Tao et al.	DIP reform and coronary heart disease costs	Interrupted time series analysis using claims data (2020–2023)	Costs and LOS declined, but divergent OOP effects by insurance type	Payment reforms improve efficiency but must be tailored to protect equity
Chang et al.	Barriers/facilitators in DIP reform implementation	Qualitative case study using the CFIR framework, based on semi-structured interviews	Leadership and guidelines facilitated reform; staff resistance and resource strain hindered	Organizational readiness and adaptive capacity are essential for reform success
Hong et al.	Cost-effectiveness of rezvilutamide in prostate cancer	Markov model-based cost-effectiveness analysis using data from the CHART phase III clinical trial	Rezvilutamide added QALYs but not cost-effective at current pricing; would be cost-effective if priced below ~$705	Pricing strategies must align innovation with affordability
Liu et al.	Digital economy and cross-border supply chain resilience	Observational provincial panel analysis (2013–2022)	Digitalization strengthened resilience via diversification and reduced concentration; strongest in west	Digital infrastructure supports resilience and sustainable access
Yang et al.	Cost-effectiveness of toripalimab in ES-SCLC	Partitioned survival model using data from the phase III EXTENTORCH trial	Cost-effective in China ($29k/QALY) but not in US ($916k/QALY)	Cost-effectiveness is context-specific, requiring alignment with local thresholds
Xu H. et al.	DIP reform and cancer costs (hematologic vs solid tumors)	Retrospective, single-center observational study	Costs declined post-reform, but hematology care exceeded reimbursement, causing deficits	Payment standards must reflect clinical complexity to ensure sustainability

VBM, value-based medicine; ICER, incremental cost-effectiveness ratio; WTP, willingness-to-pay; DRG, Diagnosis-Related Groups; TCM, Traditional Chinese Medicine; MAs: medical alliances; NVBP, National Volume-Based Procurement; QALY, quality-adjusted life year; MCDA: multicriteria decision analysis; DIP, diagnosis-intervention packet; CHD, coronary heart disease; CFIR, Consolidated Framework for Implementation Research; MAH, Marketing Authorisation Holder; ESG, environmental, social, and governance; SOE, state-owned enterprise; LOS, length of stay; ES-SCLC, extensive-stage small-cell lung cancer.

A central element of China's VBM is transparent drug pricing and reimbursement negotiations. Through the National Reimbursement Drug List (NRDL) (4), bulk purchasing has made high-cost drugs more accessible. Simultaneously, payment reforms such as the Diagnosis-Intervention Packet (DIP) system (5) bundle payments to limit unnecessary use and improve efficiency. These reforms reflect a shift away from volume-based incentives and toward structures that prioritize clinical effectiveness and sustainability.

Another frontier of innovation in VBM is the incorporation of technology-driven insights. China's experience with electronic health records, big data, and AI illustrates the potential of digital infrastructure to support outcome measurement, identify inefficiencies, and forecast future healthcare needs. These tools enable more precise, equitable, and sustainable care. For instance, AI-powered models can stratify patients by risk and guide resource allocation, ensuring that interventions generate the greatest health return per unit cost (3).

China's experience reflects global debates on sustainable healthcare financing: high-income countries face the rising costs of biologics and gene therapies, while low- and middle-income nations struggle to expand access without straining budgets. Value-based approaches offer a common framework by linking innovation, outcomes, and affordability.

This Research Topic adds valuable evidence from China, spanning hospital governance, drug pricing, supply chains, and oncology cost-effectiveness, highlighting both the promise and complexity of implementing VBM. Collectively, these studies provide a roadmap for policymakers and clinicians to balance innovation and sustainability in China and beyond.

Zhao et al. examined determinants of physicians' willingness to provide preventive services and found that intentions are shaped by norms and capacity, implying that supportive environments and digital technologies can strengthen sustainable, value-based health systems.

Chen et al. analyzed Diagnosis-Related Groups (DRG) reform effects on lumbar disc herniation patients, and found reduced hospitalization and drug costs. This study suggests that to advance VBM, payment reforms must better align with Traditional Chinese Medicine (TCM) treatment characteristics and outcome-based incentives.

Jiang et al. used interrupted time series analysis of Guangdong's insulin procurement data and found that China's National Volume-Based Procurement (NVBP) policy significantly increased insulin use while reducing total spending and unit costs. These results suggest that centralized procurement can enhance VBM by improving affordability and access without compromising treatment availability.

Xu Y. et al. established a multicriteria decision analysis framework for evaluating implantable medical devices in China, and they found that clinical safety and cost were the most influential criteria, highlighting the framework's potential to promote transparent, VBM allocation.

Wu et al. assessed the service capacity of public hospitals and found that while overall service capacity improved, disparities between hospitals widened, mainly due to inter-group differences. The authors suggest strengthening coordination, optimizing resource allocation, and improving performance evaluation to promote more balanced, value-based hospital development.

Li found that China's Marketing Authorization Holder system significantly improved pharmaceutical firms' environmental, social, and corporate governance performance by boosting investment and reducing internal inequality, supporting more sustainable, value-based industry development.

Tao et al. analyzed data from 264 hospitals and found that China's DIP reform significantly reduced hospitalization costs and length of stay for coronary heart disease patients. However, differences in out-of-pocket ratios between insurance types highlight the need to balance efficiency gains with equity in value-based payment reforms.

Chang et al. conducted qualitative interviews and found leadership support and clear guidelines that facilitated DIP reform, while resource constraints and staff resistance hindered their adoption. The study recommends strengthening internal management, communication, and staff training, underscoring that successful VBM reform requires organizational readiness and adaptive capacity alongside policy design.

Liu et al. assessed the digital economy's impact on resilience and found that digitalization strengthens resilience by reducing trade dependence, increasing export complexity, and lowering concentration. The study illustrates how digital infrastructure supports continuity of care and economic stability, extending VBM to the domain of global supply chain sustainability.

Xu H. et al. evaluated DIP reform's impact on costs for hematologic malignancies (HM). While overall costs decreased, HM treatments remained above reimbursement standards. This underscores the need to refine payment standards to reflect clinical complexity and resource intensity, ensuring departments remain financially viable.

Additionally, Zhang H. et al., Wang et al., Hong et al., and Yang et al. conducted cost-effectiveness analyses of oncology, immunotherapy, and pharmaceutical interventions, showing that clinical benefits often depend on price levels and reimbursement thresholds, underscoring the importance of price negotiation for realizing value. Zuo et al. demonstrated that cross-border knee replacement care achieved lower costs through shorter length of stay, highlighting efficiency gains from system integration. Guanyi et al. found that excessive buyer market power may suppress pharmaceutical profitability, suggesting the need to balance affordability with innovation incentives. Zhang X. et al. showed that integrating internet hospitals into medical alliances can improve referral efficiency and stakeholder value under appropriate cost-sharing arrangements, supporting value-based care by optimizing resource allocation and access across healthcare tiers.

## Conclusion

These studies show how China's healthcare reforms serve as laboratories for value-based models and illustrate the diverse domains where VBM principles apply. Key lessons include the need for equity-sensitive design, the role of technology in resilience, and the importance of aligning pricing and reimbursement with clinical and economic realities.

As health systems worldwide face rising costs and unequal access, these findings stress that value-based medicine is not monolithic but a flexible, context-sensitive paradigm. Integrating lessons from China with global best practices can help build systems that are innovative, equitable, and sustainable.
